# Supplementary material for: Exploring the textural characteristics of foods preferred by Chinese elderly individuals based on IDDSI levels
Source: PLoS One. 2025 Jan 30;20(1):e0317196. doi: 10.1371/journal.pone.0317196 (PMC11781651; doi:10.1371/journal.pone.0317196)
Supplement: Attachment — Submitted filename: 2024.11.18.docx [file pone.0317196.s006.docx]

Dear Editor,

Thank you for your review and feedback on my manuscript. I would like to clarify that my study did not involve any human remains or specimens that require special permits. Therefore, there is no need to provide additional specimen information or permit details.

To comply with the journal's requirements, I have added the following statement to the manuscript:

"No permits were required for the described study, which complied with all relevant regulations."

I have updated the manuscript accordingly and resubmitted it for your consideration. Please let me know if there are any further questions or clarifications needed.

Thank you once again for your attention to my manuscript and your valuable suggestions.

Best regards,

Muxi Chen
