# Supplementary material for: Exploring the textural characteristics of foods preferred by Chinese elderly individuals based on IDDSI levels
Source: PLoS One. 2025 Jan 30;20(1):e0317196. doi: 10.1371/journal.pone.0317196 (PMC11781651; doi:10.1371/journal.pone.0317196)
Supplement: Attachment — Submitted filename: Response to Reviewers.docx [file pone.0317196.s007.docx]

Dear Editor and Reviewers,

We sincerely appreciate the time and effort you have invested in reviewing our manuscript and providing valuable feedback. We have carefully considered each comment and made the corresponding revisions to improve the clarity, accuracy, and overall quality of our work. Below, we detail our point-by-point responses to Reviewer #5’s comments and the specific changes implemented in the revised manuscript. Revised sections are clearly indicated in the updated version.

**Reviewer #5’s Comments and Our Responses**

**1.Texture Measurement Methods (Section 2.5) - Instrument Make and Model**
**Comment:** In Section 2.5, please provide the make and model of the texture analyzer used.
**Response and Revision:** We have added the specific make and model of the instrument. The text now reads: “Texture measurements were performed using a texture analyzer TA.new plus (ISENSO, USA).”

**2.Repetition in Food Processing Description (Section 2.3)**
**Comment:** The egg-to-water ratio (1:2) is repeated multiple times, though it is clearly stated in the first paragraph of Section 2.3. Avoid unnecessary repetition.
**Response and Revision:** We have removed all redundant mentions of the egg-to-water ratio, retaining only the initial statement in the first paragraph of Section 2.3

**3.Wording and Unit Standardization**
**Comment:** On page 56, “A research supports” should be changed to “Previous research supports.” Also, please use “mL” instead of “ml.”
**Response and Revision:** We have revised the sentence to “Previous research supports…” and have standardized all volume units to “mL” throughout the manuscript.

4.**Unit for Cohesion in Table 6**
**Comment:** Specify the unit for Cohesion in Table 6 (e.g., gf).
**Response and Revision:** We have updated Table 6, now labeling the Cohesion column as “Cohesion (gf).”

**5.Predictive Equation Clarification**
**Comment:** The predictive equation should include a clear explanation of where the values (e.g., intercepts from multiple linear regressions) are derived, rather than relying on the reader’s memory.
**Response and Revision:** We have revised the accompanying text for the predictive equation, explicitly stating that the intercepts and parameters (e.g., adhesion, cohesion) originate from the multiple linear regression analyses presented in Table 6. This clarification allows readers to fully understand the source of these values without referring back to earlier sections.

**6.Figure Explanations and Color Improvements**
**Comment:** The figures lack sufficient explanation, and the colors are difficult to distinguish. Each figure should provide more context. For example, Fig. 1 should explain the scope of hardness for the seven food types and indicate peaks. Similarly, Fig. 2 and Fig. 3 need more descriptive captions. Please also address the color contrast issues.
**Response and Revision:**

We have expanded and clarified the figure captions as follows:

**Fig. 1 (Revised Caption):** “This figure illustrates the hardness profiles of seven food categories. Each color corresponds to a distinct food group. Solid and dashed lines represent the maximum and minimum values, respectively, while the shaded area indicates the overall range. Peaks and distributions of hardness for each category are highlighted to facilitate comparisons.”

**Fig. 2 (Revised Caption):** “This figure shows the cohesiveness distributions of seven food groups across different IDDSI levels. Each color represents a distinct category. Solid and dashed lines indicate the maximum and minimum values, and the shaded area represents the range. Overlapping distributions highlight the complexity of categorizing these foods within IDDSI levels.”

**Fig. 3 (Revised Caption):** “This figure displays the adhesiveness values of seven food categories. Each color corresponds to a different group, with solid and dashed lines marking maximum and minimum values, and the shaded area showing the range. The figure reveals distinct peaks in adhesiveness, enabling straightforward comparisons among the categories.”

To address color differentiation issues, we have selected seven colors with higher contrast and employed a combination of solid and dashed lines, as well as shaded areas, to represent maximum, minimum, and range values. This approach ensures that readers can easily distinguish between categories, even when viewed in grayscale.

We thank you again for your constructive and insightful feedback. We believe that these revisions have substantially improved the manuscript’s clarity, coherence, and readability. If you have any further comments or suggestions, we will be happy to

address them.

Sincerely,
Chen Muxi
