# Supplementary material for: Exploring the textural characteristics of foods preferred by Chinese elderly individuals based on IDDSI levels
Source: PLoS One. 2025 Jan 30;20(1):e0317196. doi: 10.1371/journal.pone.0317196 (PMC11781651; doi:10.1371/journal.pone.0317196)
Supplement: Attachment — Submitted filename: Reviewer Comments.docx [file pone.0317196.s005.docx]

Dear Editor,

I would like congratulate the authors for producing good quality manuscript, but I have some queries and suggestions to increase the quality of the manuscript:

1. Kindly mention the study design of this manuscript.
2. Paired t was used in the study. It is a parametric test, was the normality of the data checked before applying this test. If so kindly mention.
3. Kindly upload high resolution images for figures.
4. Abstract is supposed to provide the gist of the manuscript. Kindly reduce the word count of your abstract.
5. Discussion should be less quantitative, kindly reduce the use of data in the discussions section and compare it with recent literature.
6. Discussion section can be elaborated after a thorough literature review.
7. Kindly provide the strengths and limitations of this study.
8. Kindly mention the future recommendations for this study
